# “To teach or not to teach- that is the question” The educational and clinical impact of introducing an outcome based, modular curriculum in Social Emergency Medicine (SEM) at a private tertiary care center in Karachi, Pakistan

**DOI:** 10.1186/s12909-023-04385-z

**Published:** 2023-06-10

**Authors:** Saima Ali, Syed Ghazanfar Saleem, Adeel Khatri, Sama Mukhtar

**Affiliations:** Department of Emergency Medicine, Indus Hospital and Health Network, Karachi, Pakistan

**Keywords:** Emergency department, Social determinants of health (SDH), Social emergency medicine (SEM), Curriculum, Bounce-back

## Abstract

**Introduction:**

An enhanced knowledge of Emergency Medicine (EM) personnel regarding negative Social Determinants of Health (SDH) can impact EM service provision in a resource limited country like Pakistan. Interventions to build capacity in identifying and addressing these SDH through education in Social Emergency Medicine (SEM) can be one of the ways in which EM key performance indicators (KPIs) can be improved.

**Method:**

A SEM based curriculum was administered to the EM residents at a tertiary care center in Karachi, Pakistan. Pre, post and delayed post-test was conducted for knowledge of EM residents and analyzed using Repeated Measures ANOVA (RMANOVA). Clinical impact of this intervention was assessed through the ability of the residents to identify the patients’ SDH and determining appropriate disposition. Comparison of the bounce-back of patients in the pre-intervention (2020) and post-intervention year (2021) year was appreciated to see the clinical impact of this intervention.

**Result:**

A significant improvement was seen in post intervention (*p* < 0.001) and follow up knowledge (*p* < 0.001) of residents regarding negative SDH. Bounce-back rate was higher in the pre-SEM curriculum (43%) as compared to the post-SEM curriculum year (27.7%). Post-intervention, the residents were able to identify the unique Pakistani SDH, however appropriate patient disposition needs further reinforcement.

**Conclusion:**

The study highlights the beneficial impact of an educational intervention in SEM upon the knowledge of EM residents and the bounce-back of patients in the emergency department (ED) of a low resource setup. This educational intervention can be scaled up to other EDs across Pakistan for potential improvement in knowledge, EM process flow and KPIs.

**Supplementary Information:**

The online version contains supplementary material available at 10.1186/s12909-023-04385-z.

## Introduction

“Medicine has imperceptibly led us into the social field and placed us in a position of confronting directly the great problems of our society.” Rudolph Virchow

Research estimates that 50% of health outcomes are shaped by social and economic factors [1]. Addressing the 3Ds; social determinants, health disparity and health-care workforce diversity through well-defined educational interventions has been the subject of interest for many years. Holistic patient care mandates a dual medical and social approach through incorporation of structured education on SDH in the under-graduate and post-graduate medical curricula [2, 3]. The need for inter-professional-education (IPE) has also been advocated for better liaison between medical work force in hospitals and community-based support networks to address the negative SDH that plague the smooth functionality our healthcare systems [4, 5].

Various articles in under-graduate medical education have emphasized upon SDH centered education in both pre-clinical and clinical years, using case-based teaching with assessment [6]. Application of a structural competency frame work as a theory-based guide to teach advocacy was also found helpful in enabling under-graduate medical students in identifying factors that influence health inequities [7]. Support from course directors, administrators and faculty leadership in pre-clinical content generation and developing cross-curricular integration were considered essential in developing a successful under-graduate curriculum addressing negative SDH [8]. In Pakistan, various efforts have been made to teach humanities and social sciences in under-graduate medical education (UGME) to develop awareness about SDH. From the well-received and implemented humanities and social sciences (HASS) curriculum being offered at a private medical college to the troubled community oriented medical education (COME) curriculum introduced in public medical colleges [9], these efforts have been partially successful. Barriers such as absence of needs assessment, lack of institutional preparedness and buy in from the stake holders, led to the decline of the COME program. This emphasized that gradual phase in of the curricular change, involvement of the stake holders from the planning phase, sustainability of the teaching faculty and resources with legislative support are essential to ensure that such a curricular innovation is met with success [10].

SDH are seen at play every day in EDs across the globe, where patients with diverse demographic and socioeconomic background seek care. Many of these patients lack the resources and insight into their neglected medical issues. They may also be seeking refuge from environmental extremes, or because they are unable to deal with their SDH. Several post-graduate residency programs globally use well defined curricula to address SDH along with proper assessment and feedback methodologies [11] and integration of SDH-based EM clerkship of under-graduate medical students has been suggested [12]. There is, however, a dearth of clear delineation of problems, systematic interventions, collaboration and advocacy of policies to improve the outcome of these ED patients [13].

The amalgamation of social medicine into EDs has been fairly recent and the term “Social Emergency Medicine (SEM)” was coined in 2009 in the USA with the concept that EDs can address the negative SDH of patients while providing resource intensive, critical care [14]. To address issues like discrimination, neglect and barriers to seeking healthcare, ED at Highland Hospital in Oakland, California, established “The Andrew Levitt Center for Social Emergency Medicine”, leading to the education of SEM at the post graduate level [14]. In 2017, the American College of Emergency Medicine (ACEP) established their SEM section and the social medicine and population health interest group was started at the Society of Academic Emergency Medicine (SAEM) [15]. This paved the way to fellowships in SEM in the USA, that focus on incorporation of the patients’ social context into ED management, promote research into SDH, apply research for best practice in patient care with evaluation and critique of health policies that affect SDH.

The role of EM physicians in low-and-middle-income countries (LMIC) like Pakistan is impacted by universal negative SDH such as homelessness/ unstable housing, food insecurity, substance abuse, community/ domestic/ intimate partner violence and health literacy. Many factors unique to Pakistani culture like myths surrounding communicable diseases, traditional/ faith healers, local practices that deter early diagnosis and treatment of illness, taboos surrounding sexual health and trans-genders, teenage marriages and pregnancies, multi-parity and contraception are found to be at play. These issues, when not addressed in a timely fashion, add to the cost and disease burden for patients and their families, as was identified at the Indus Hospital and Health Network (IHHN) in Pakistan, that provides free health-care delivery, rooted in philanthropy, in fifteen hospitals across the country. Due to the free-for-service-model, IHHN caters largely to the marginalized and under-privileged members of the society. The EM residents, enrolled for post-graduate training by the College of Physicians and Surgeons, Pakistan (CPSP) at IHHN were found to lack knowledge about the negative SDH of their patients. This was identified as one of the reasons contributing to the recurrent presentation of the patients with the same pathology (bounce-back).

To address this issue, a one-month module in SEM was introduced to the EM residents at IHHN. The SEM module was based on themes, focused on negative SDH and was developed keeping the contextual SDH of Pakistan in mind. The goal was incorporating social consideration in patient management with the prospect of developing holistic EM physicians with the ability to utilize available resources for the advantage of the patients and their care givers. EM physicians were also encouraged to overcome their own implicit and explicit biases in managing patients with negative SDH [16]. The over-arching future aim was singling out the champions for the cause of SEM and development of sustainable programs that address the negative SDH.

After the administration of the teaching module on SEM, this study was conducted at IHHN to evaluate the impact of the SEM education on the EM residents’ knowledge. Another objective was to assess whether the ability to identify the patients’ negative SDH can lead to their proper disposition with a change in the rate of their bounce-back visits. The overarching goal was an improvement in the patients’ clinical outcome and the ED process flow.

## Materials and method

### Study site and participants

After approval from Institutional Review Board (IRB) at IHHN, the study was conducted at the IHHN ED in Karachi, Pakistan. The study was conducted over four months from 1^st^ March 2021 to 30^th^ June 2021. All twenty EM residents were included after taking written, informed consent and there was no student drop-out during the period of the study.

### Study design

A Quasi-experimental study deign was chosen because of the intervention of SEM curriculum.

### Curriculum development of the educational intervention

By virtue of being a member of the ACEP SEM section, the principal investigator (PI) who is a Pakistani EM clinician educator, made a formal request via e-mail to a USA based post-graduate SEM fellowship program to share their curricula. The curriculum is open access and written permission was taken to use the themes identified in that curriculum, as a reference. The IHHN EM SEM curriculum was developed using Kern’s six steps of curriculum development. These included problem identification, general and targeted needs assessment, setting of goals and objectives, developing an educational strategy, modular implementation and getting evaluation and feedback.

Needs assessment (both general and targeted) was conducted to identify the stake holders of the SEM curriculum (Table 1) as it is a relatively novel concept in EM in Pakistan and is not a part of the formal national EM residency curriculum.

**Table 1:** SEM needs assessment

Learner (EM Residents)	Environment (Emergency Department)	Stake holders (IHHN, PGME, CPSP)
First-hand management of the patients presenting to ED due to SDH	Inclusiveness of the marginalized population at IHHN	Curriculum can be scaled up to involve other specialties across the hospital and other EDs across the country
High bounce-back to ED due to negative SDH	Community-based programs already in existence	CPSP currently has no curriculum that addresses SDH
Awareness of available community and social service support	Judicious use of health-care resources (custodians of zakat and donation)	

### SEM Entrustable Professional Activities (EPAs)

A modular, competency-based curriculum, with entrustable professional activities (EPAs) grounded in the Canadian Medical Education Direction for Specialists (CanMEDS) competencies was developed by the PI (Annexure 1). The competencies included medical expert, communicator, collaborator, scholar, leader, healthcare advocate and professional. The EPAs were executable within a given time, observable, measurable and suitable for focused entrustment decisions and were further broken down into themes that were rooted in SDH (Annexure 2).

### SEM Educational strategy

Education in the SEM module was over one month (from 1^st^ March 2021 to 31^st^ March 2021) with two classes per week, each of two hours duration, following all social distancing standard operative procedures. There was a total of 19 h interaction with each resident. One month teaching module with two hour, bi-weekly sessions (sixteen hours), pre and post-test (one hour each) and delayed post-test for retention of knowledge, four months after intervention in August 2021 (one hour). Twenty EM residents, registered with the post-graduate medical education office were included through non-probability, purposive sampling and were administered the SEM curriculum. Facilitators for the curriculum were EM physicians, medical educators, legal experts and social workers who had a minimum of ten years’ experience of managing SDH in the Pakistani health-care sector. The facilitators were briefed prior to each session and the core faculty for the module co-facilitated each session. Pre-reading resources were shared with the residents a week ahead.

A SEM theme was introduced at the beginning of the day along with the learning objectives, inviting discussion from the residents and answering their queries. This was followed by case based, small group discussion and simulation, based on concepts of SEM. This was further augmented through bedside teaching and chart simulated recall. Residents were encouraged to document the identified patients’ SDH in electronic health record (EHR) during their clinical practice and mention the way it was addressed.

Formative assessment was provided using the Mini-CEX and chart simulated recall. Summative assessment of the module was based on a written, knowledge based MCQ examination as pre & post-test with delayed post-test, conducted four months after the intervention. A plan for assessment of individual SEM based competencies was developed for future use.

### Data collection

The examinations were paper based MCQs, forty questions each, administered over one hour and were targeted to assess SEM concepts. The results were saved for analysis under password protection. As IHHN is a paperless hospital, EHR was reviewed to collect retrospective patient data from 1^st^ April to 30^th^ June 2020, prior to the introduction of the SEM intervention. Patient data was also collected from the EHR post SEM intervention from 1^st^ April to 30^th^ June 2021. The data was collected for all times of day and night. In the post SEM intervention, documented SDH were noted for the index visit from the EHR. Data set was obtained for the bounce-back of patients at 24, 48 and 72 h by review of Manchester Triage System, a five-level system, used for triaging patients at IHHN ED, that was accessible to the PI.

General demographic information of the patient; age, gender, presenting complaint and disposition (admitted, referred, discharged, expired) was also recorded. All the information was entered on a pre-designed questionnaire.

### Ethical considerations

All the residents were educated in the SEM module and there was no randomization. IRB approval was taken for the study. Informed written consent was sought by the PI from the residents in person at IHHN, Karachi, on a paper based, English consent form with signature. Any queries raised during the obtaining of consent were answered. Identification of the participants was kept confidential through codes with issuance of unique identity. Electronic data was password protected and was accessible only to the PI and research associate. After the publication of the study, the data will be archived under password protection. The IHHN seven-year data retention policy shall be followed.

### Data analysis

The data was entered and analyzed on IBM, SPSS version 21 and R version 4.2.2 (2022–10-31). Paired sample t-test was used to observe any significant difference between pre and post intervention knowledge of the residents. Repeated Measure Analysis of Variance (RMANOVA) was applied to determine the significant difference in pre, post and delayed post-test for knowledge of residents about SEM curriculum. P-value of ≤ 0.05 was considered statistically significant.

Continuous data of patients’ age was observed for pediatric, adult and geriatric observations, while frequency with percentage were obtained for categorical data like patients’ disposition and number of times of bounce back. The association between the negative SDH, year of residency and the disposition of the patients was also recorded using a two-way table in R studio.

## Results

Sixteen residents were enrolled in the residency program in 2020 and four more enrolled in 2021, making a total of twenty residents who underwent the SEM educational intervention, thirteen (65%) of whom were women and seven (35%) were men (Table 2).

**Table 2:** Demographic details of the EM residents (2020 & 2021)

Resident gender	Year	
	**2020**	**2021**
**Men n (%)**	6 (37.5%)	7 (35%)
**Women n (%)**	10 (62.5%)	13 (65%)
**Total Number of Residents (n)**	16	20
**Residents’ Distribution According to Year of Residency**		
**Year 1**	7	4
**Year 2**	4	7
**Year 3**	5	4
**Year 4**	0	5

A significant mean difference (*p* < 0.001) was observed between the pre (19.8 ± 3.9) and post intervention knowledge (31.4 ± 4) of the residents using the student t-test. Pre, post and delayed post-test knowledge were also significantly different in residents (*p* < 0.001) in the post intervention and follow up knowledge, using the Repeated Measures ANOVA (RMANOVA) (Table 3).

**Table 3:** Pre, post and delayed-post test mean difference in knowledge of EM residents who underwent the SEM educational intervention (2021)

**Mean Difference of Pre and Post Test using student t-test**			
	Mean ± SD	t statistics	*P* value
Pre-test	19.8 ± 3.9	-13.6	< 0.001
Post-test	31.7 ± 4		
**Mean Difference of Pre, Post and Delayed Post-Test using RMANOVA**			
	Mean ± SD	f statistics	*P* value
Pre-test	19.8 ± 3.9	5057.1	< 0.001
Post-test	31.7 ± 4		
Delayed post-test	30.1 ± 2.5		

The EM residents attended a total of 6607 patients in the study period out of which 1749 (43%) patients bounced-back in the year 2020 and 704 (27.7%) bounced-back in the year 2021 (Table 4). The highest number of bounce-back patients was seen by year-one EM residents across both 2020 (51.11%) and 2021 (32.67%), with majority of the patients presenting back within 24 h of their index visit.

**Table 4:** Frequency of bounce back patients with year of residency (2020 and 2021)

**Year**	**2020**				**2021**			
**Total Bounce Back Patients n (%)**	**1749 (43%)**				**704 (27.7%)**			
**Bounce back (hours)**	**24**	**48**	**72**	**Total Patients**	**24**	**48**	**72**	**Total Patients**
**Bounce back Patients per Year of Residency n (%)**								
**Year 1**	781 (44.65)	101 (5.77)	12 (0.68)	894 (51.11)	209 (26.68)	20 (2.84)	1 (0.14)	230 (32.67)
**Year 2**	437 (24.98)	52 (2.97)	6 (0.34)	495 (28.30)	178 (25.28)	12 (1.70)	3 (0.42)	193 (27.41)
**Year 3**	312 (17.83)	44 (2.51)	4 (0.22)	360 (20.58)	130 (18.46)	10 (1.42)	1 (0.14)	141 (20.02)
**Year 4**	0 (0)	0 (0)	0 (0)	0 (0)	132 (18.75)	6 (0.85)	2 (0.28)	140 (19.88)

The highest number of bounce-back patients across both years were between 15–59 years of age, with 77.93% patients in 2020 and 71.44% patients in 2021 with more women presenting in 2021 as compared to 2020 (60.36% versus 40.25%) (Table 5).

**Table 5:** Demographic details of the bounce-back patients (2020 and 2021)

**Year**	**2020**	**2021**
**Total Patients n (%)**	1749	704
**Age (years)**		
** < 14**	165 (9.43%)	97 (13.77%)
**15–59**	1363 (77.93%)	503 (71.44%)
** > 60**	221 (12.63%)	104 (14.77%)
**Gender**		
**Men**	1045 (59.74%)	279 (39.63%)
**Women**	704 (40.25%)	425 (60.36%)

The pre-dominant final disposition of the bounce-back patients in both years was either discharge or referral to other facilities due to non-availability of beds or required specialty service. This disposition pattern was similar across all age groups and years of residency, however patients who expired in 2021 were less as compared to 2020 (Tables 6 and 7).

**Table 6:** Pre-intervention disposition of bounce-back patients according to age group and year of residency (2020)

**Disposition**	**Patients’ age group n (%)**			**Year of residency n (%)**		
	** < 14**	**14–59**	** > 60**	**1**	**2**	**3**
**Admitted**	7 (0.40)	36 (2.05)	3 (0.17)	22 (1.25)	16 (0.91)	8 (0.45)
**Discharged**	50 (2.85)	373 (21.32)	41 (2.34)	245 (14.00)	130 (7.43)	89 (5.08)
**Expired**	15 (0.85)	163 (9.31)	43 (2.45)	108 (6.17)	66 (3.77)	47 (2.68)
**LAMA**	7 (0.40)	52 (2.97)	7 (0.40)	31 (1.77)	19 (1.08)	16 (15.09)
**Referred out**	86 (4.91)	739 (42.25)	127 (7.26)	488 (27.90)	264 (15.09)	200 (11.43)

**Table 7:** Post-intervention disposition of bounce-back patients according to age group and year of residency (2021)

**Disposition**	**Patients’ age group n (%)**			**Year of residency n (%)**			
	** < 14**	**14–59**	** > 60**	**1**	**2**	**3**	**4**
**Admitted**	2 (0.28)	26 (3.69)	9 (1.27)	16 (2.27)	10 (1.42)	4 (0.56)	7 (0.99)
**Discharged**	85 (12.07)	335 (47.58)	54 (7.67)	147 (20.88)	136 (19.31)	94 (13.35)	97 (13.77)
**Expired**	0 (0)	12 (1.70)	14 (1.98)	7 (0.99)	6 (0.85)	6 (0.85)	7 (0.99)
**LAMA**	6 (0.85)	61 (8.66)	6 (0.85)	25 (3.55)	15 (2.13)	13 (1.84)	20 (2.84)
**Referred out**	4 (0.56)	66 (9.37)	21 (2.98)	35 (4.97)	25 (3.55)	23 (3.26)	8 (1.13)
**Sent for Medicolegal**	0 (0)	3 (0.46)	0 (0)	0 (0)	1 (0.14)	1 (0.14)	1 (0.14)

Although the total number of bounce-back patients was less in 2021, among all the bounce-back patients in the years 2020 and 2021; the highest number of patients presented during the first 24 h of their index visit. The numbers of the P1 (emergent acuity) bounce-back patients within 24 h remained almost the same in both years (6.17% in 2020 versus 6.67% in 2021) however the number of the P2 acuity (very urgent) bounce-back patients reduced from 35.96% in 2020 to 23.72% in 2021. A rise in the numbers of P3 and P4 (urgent and standard) patients was observed across both years in their first bounce-back visit within 24 h; 40.59% in 2020 versus 54.54% in 2021 for P3 and 3.83% in 2020 versus 7.24% in 2021 for P4 respectively. The overall numbers of patients presenting within 48 to 72 h were low in 2021 as compared to 2020 (Table 8).

**Table 8:** Bounce-back frequency with acuity at index visit

**Bounce-back numbers n (%)**	**Bounce-back frequency (hours)**					
	**2020 (1749)**			**2021 (704)**		
	**24**	**48**	**72**	**24**	**48**	**72**
	1530 (87.47)	197 (11.26)	22 (1.25)	649 (92.18)	48 (6.81)	7 (0.99)
**Acuity at Index Visit**						
**P1**	108 (6.17)	17 (0.97)	0 (0)	47 (6.67)	0 (0)	0 (0)
**P2**	629 (35.96)	77 (4.40)	10 (0.57)	167 (23.72)	5 (0.71)	1 (0.14)
**P3**	710 (40.59)	92 (5.26)	12 (0.68)	384 (54.54)	35 (4.97)	6 (0.85)
**P4**	67 (3.83)	7 (0.40)	0 (0)	51 (7.24)	8 (1.13)	0 (0)
**P5**	16 (0.91)	4 (0.22)	0 (0)	0 (0)	0 (0)	0 (0)

**Fig. 1:**
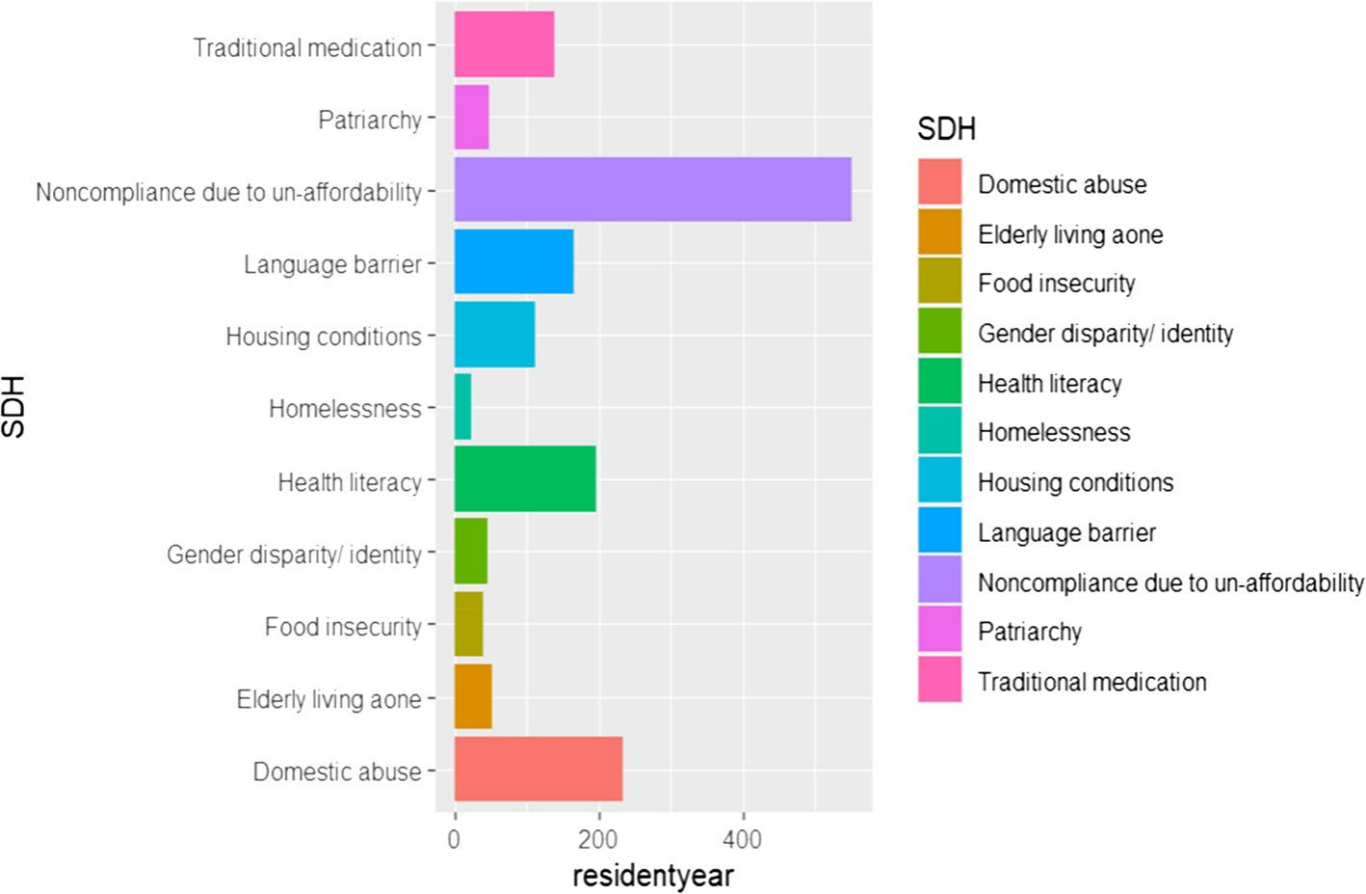
SDH identified in the bounce-back patients

The research established that noncompliance to medication due to unaffordability was the major SDH, seen in 254 patients (36.1%), followed by domestic abuse in 97 (13.8%), lack of health literacy in 86 (12.2%) and lack of health provision due to language barrier in 68 (9.6%) patients respectively (Fig. 1).

A look at the disposition of the bounce-back patients with their identified SDH and the year of residency revealed that although domestic abuse was identified by residents across all years, the disposition was not appropriately decided and most of these patients were discharged (5.25%) or left-against-medical-advice (LAMA) (7.24%), instead of being referred for medicolegal help (ML) (0.42%). Those who had bounced-back due to inappropriate counselling resulting from language barrier, were appropriately discharged (8.94%) and the disposition was similar in those who presented due to lack of health literacy (7.52%) (Table 9).

**Table 9:** Relationship of bounce-back patients’ sdh with disposition and year of residency

**SDH**	**Year of resideny n (%)**				**Disposition n (%)**					
	**1**	**2**	**3**	**4**	**Admitted**	**Discharged**	**Expired**	**LAMA**	**Referred out**	**Referred for ML**
Domestic Abuse	28 (3.97)	28 (3.97)	15 (2.13)	26 (3.69)	2 (0.28)	37 (5.25)	0 (0)	51 (7.24)	4 (0.56)	3 (0.42)
Elederly living Alone	3 (0.42)	9 (1.27)	5 (0.17)	4 (0.56)	2 (0.28)	15 (2.13)	0 (0)	0 (0)	4 (0.56)	0 (0)
Food Insecurity	5 (0.71)	2 (0.25)	5 (0.17)	4 (0.56)	0 (0)	13 (1.84)	0 (0)	0 (0)	3 (0.42)	0 (0)
Gender Disparity	8 (1.13)	6 (0.85)	3 (0.42)	4 (0.56)	0 (0)	16 (2.27)	1 (0.14)	4 (0.56)	0 (0)	0 (0)
Health Literacy	31 (4.40)	19 (2.69)	18 (2.55)	18 (2.55)	9 (1.27)	53 (7.52)	6 (0.85)	4 (0.56)	14 (1.98)	0 (0)
Homelessness	0 (0)	4 (0.56)	2 (0.28)	2 (0.28)	0 (0)	6 (0.85)	0 (0)	0 (0)	2 (0.28)	0 (0)
Housing Conditions	16 (2.27)	11 (1.56)	11 (1.56)	10 (1.42)	3 (0.42)	41 (5.82)	0 (0)	3 (0.42)	1 (0.14)	0 (0)
Language Barrier	21 (2.98)	17 (2.41)	11 (1.56)	19 (2.69)	1 (0.14)	63 (8.94)	0 (0)	1 (0.14)	3 (0.42)	0 (0)
Non-compliance due to Unaffoardibility	86 (12.21)	77 (10.93)	53 (7.52)	38 (5.39)	16 (2.27)	189 (26.84)	14 (1.98)	5 (0.71)	30 (4.26)	0 (0)
Patriarchy	8 (1.13)	7 (0.99)	3 (0.42)	4 (0.56)	0 (0)	9 (1.27)	0 (0)	5 (0.71)	8 (1.13)	0 (0)
Traditional Medication	24 (3.40)	13 (1.84)	15 (2.13)	11 (1.56)	4 (0.56)	32 (4.52)	5 (0.17)	0 (0)	22 (3.12)	0 (0)

## Discussion

From the most commonly understood role of providing healthcare to the seriously ill and injured patients, to being the only door open to the marginalized, EDs serve as a safety net for the entire healthcare system [17]. Our study showed an improvement in the knowledge of EM residents regarding the negative Pakistani SDH and ways to address them, highlighting the importance of structured teaching of SDH. This is similar to the studies found in literature that have signified that modular teaching of SDH through a SEM curriculum can have a far greater impact on the resident knowledge and perception [18]. However, research on such curricular interventions has not compared knowledge results, pre and post intervention and is generally qualitative.

In a recently published scoping review, a comprehensive literature appraisal was done that emphasized ED based interventions and their impact on SDH [19]. It highlighted the studies that focused on educating EM residents and allied health-care professionals about SDH and categorized the types of educational interventions, SDH domains and outcome. However, no studies were identified till date on teaching a structured SEM curriculum in an EM residency program with assessment of knowledge and its suggested impact on ED quality metrices such as patient bounce-back. Although other factors like resident seniority and COVID-19 might have also influenced the rate of patient bounce-back, a slight improvement in this matric is a promising reflection upon the possibility of exploring this further.

Our novel study in the Pakistani context can be considered a first-step towards sensitization of EM physicians to the negative SDH and developing mental maps to appropriately manage them. Designing ED based social intervention that can improve patients’ disposition and outcome is an uphill task in LMIC like Pakistan where the access to basic health-care is limited. Addressing issues like domestic violence, homelessness, gender disparity due to cultural norms and patriarchy, require mobilization of resources both in the health-care sector and the community [20]. Our educational intervention on SEM can be used as a model to design contextual curricula in other resource limited countries with similar negative SDH and can provide a roadmap towards addressing issues like compromised process flow in a busy ED.

The incidence of bounce-back varies globally between 1.1 to 33% in the USA, 4.5 to 8.7% in Canada, 1.9 to 15.8% across Europe and 5% in Australia [21]. Multivariate analyses have shown 72-h ED bounce back in elderly men and those with advanced age, higher triage acuity, language barrier (in terms of English proficiency), lack of insurance, chronic illness/ illness severity and co-morbidities [22, 23]. There is evidence that increased number of bounce-back visits are associated with an increase in five-year mortality of patients [24] and a similar high mortality was noted in our bounce-back patients although identifying and addressing the social issues at the index visit might have led to fewer patients presenting again. The influence of the initial disease process and management of the patients upon the outcome is significant, however, the positive effect of the SEM curriculum upon preventable mortality of bounce-back patients was evident. In view of this finding, there is a need to upgrade the triage scoring once such patients present to the ED to cater to their specific needs.

It was also observed that majority of the bounce-back patients were seen by junior residents, with a significantly worsening acuity on bounce-back This indicates that clinical experience of the senior residents, resulted in addressing the problems at the patients’ index visit. This is inconsistent with the findings in a recent study that suggests that the rate of bounce-back is similar across residency years [25]. The reason for the difference might be the constant exposure of the residents at IHHN to socially marginalized patients. This has provided data that clinical supervision of residents has to be more vigilant with a sign-off by the attending physician, which would improve their clinical performance. Such standards have not been set by CPSP or assessed in the form of workplace-based assessment (WPBA) in EM. In order to improve upon the ED process flow, establish a culture of patient safety and improve ED quality of service, such measures are the need of the day in EDs across Pakistan.

### Limitations

This study was conducted in one non-public sector health network, which was free for service and rooted in philanthropy. The results may not be generalizable to other public or private sector institutions. Similarly, the study did not take into account index visit misses that never came back to the ED. ED length of stay was not studied, but it is related to bounce-back and patient disposition which was studied and documented. This study was conducted during the first wave (2020) and third wave (2021) of COVID-19 and a lot of patients were bouncing back due to COVID and its related medical and socioeconomic complications.

## Conclusion

The importance of SDH and their impact on the provision of emergency medical care cannot be emphasized enough. The use of structured teaching in SEM is the first step towards moving away from the traditional model of ED care to a model that is more humane. Using the experience in teaching and learning SEM, it is hoped that this curriculum is incorporated in the current CPSP EM curriculum. It is, therefore, imperative that this study is followed up by further studies to look at the role of EM in addressing these negative SDH through identification of local champions and strengthening ties with primary care and the community.

## Supplementary Information

**Additional file 1:** **Annexure 1. **SEM Entrustable Professional Activities(The outcomes and objectives are selective for the sake of summation). **Annexure 2. ** Thematic Breakdown of SEM Curriculum (Only one theme discussed).

## Data Availability

The datasets used and/or analyzed during the current study are available from the corresponding author upon reasonable request.
