# Supplementary material for: “To teach or not to teach- that is the question” The educational and clinical impact of introducing an outcome based, modular curriculum in Social Emergency Medicine (SEM) at a private tertiary care center in Karachi, Pakistan
Source: BMC Med Educ. 2023 Jun 10;23:429. doi: 10.1186/s12909-023-04385-z (PMC10257367; doi:10.1186/s12909-023-04385-z)
Supplement: Supplementary file 1 — Additional file 1: Annexure 1. SEM Entrustable Professional Activities(The outcomes and objectives are selective for the sake of summation). Annexure 2. Thematic Breakdown of SEM Curriculum (Only one theme discussed). [file 12909_2023_4385_MOESM1_ESM.docx]

**ANNEXURES:**

**Annexure 1: SEM Entrustable Professional Activities (The outcomes and objectives are selective for the sake of summation)**

| **Medical Expert:**  -Residents should have updated knowledge of relevant biomedical, clinical, epidemiological and social-behavioral sciences related to SDH and ability to apply this knowledge to patient care in varied clinical and community settings.  -The residents must apply principles of evidence-based medicine for the provision of efficient, effective and compassionate patient care that encompasses both treatment of illnesses and promotion of health and creates awareness about and improvement in SDH of their patients. | | |
| --- | --- | --- |
| **Year 1-3** | **Year4** | **Year 5** |
| **Outcome/ EPA 1: Perform SDH Focused History and Physical Examination** | | |
| Synthesize essential data necessary for the correct management of a patient with multiple SDH. | Identify life-threatening conditions and the most likely diagnosis based on history and physical examination. | Co-relate essential patient data using all available data sources to manage the patient’s SDH. |
| **Outcome / EPA 2: Recognize Cultural Differences and Their Impact on Healthcare** | | |
| Understand the connection between culture and healthcare. | Recognize and reconcile socio-cultural differences between healthcare provider and patient to achieve patient centric care. | Use the five components of communication; repertoire, situational awareness, adaptability and knowledge of core cultural issues for empathetic patient care. |
| **Outcome / EPA 3: Understand Race as a Social Construct and How Racial Bias Contributes to Health Disparity** | | |
| Understand healthcare providers implicit racial biases and the means to counter them. | Address racial biases in day-to-day patient care, comprehending that racial bias is not only dependent on skin color. | Increase awareness of how racial bias can affect patient care and apply that knowledge in teaching junior colleagues. |
| **Scholar:**  The residents must demonstrate habitual and judicious use of SDH based communication, knowledge, technical skills, clinical reasoning, emotional (cultural and social) values and reflection in daily practice for the benefit of the individual and community being served. | | |
| **Outcome / EPA 1: Demonstrate Optimal Patient Care through Evidence-Based Practice and Continuous Learning about SDH and their Application in ED** | | |
| Perform specific literature search into SDH in ED. | Demonstrate evidence-based practice in clinical work, rooted in principles of SDH. | Plan, participate in and conduct research activities in developing interventions to address SDH. |
| **Outcome/EPA 2. Teach Colleagues in a Safe Learning Environment through Disseminating Knowledge, Instructing Skills and Providing Feedback of their Performance on Addressing SDH in ED** | | |
| Refer to guides and frameworks in managing SDH. | Ensure safe learning environment in their own practice and teaching junior colleagues about SDH. | Provide constructive feedback in teaching and learning activities focused on SEM. |
| **Communicator:**  The residents should develop interpersonal and communication skills to address SDH that will result in effective exchange of information with patients, their families and professional colleagues. | | |
| **Outcome/ EPA 1: Demonstrate the Importance of Healthcare Provider Communication in Improving Health Literacy for Patients** | | |
| Ensure that patients and their care givers understand medical instructions through improving health literacy. | Identify the need of trained interpreters and mobilization of resources. | Ensure continuity of care by developing materials like pre-printed discharge instructions. |
| **Outcome/ EPA 2: Communicate Effectively (Written and Verbal) with Colleagues, Patients and Families from a Broad Range of Socioeconomic and Cultural Background** | | |
| Use patient-centered interviewing skills to effectively gather relevant biomedical and psychosocial information. | Demonstrate clear communication with patients and their families during interactions at different times. | Share information and explanations that are clear, accurate, and timely, while checking for comprehension |
| **Collaborator:**  The residents should work effectively with other health-care professionals and ancillary healthcare departments (e.g patient welfare, NGOs, Department of safety and security, law enforcement agencies etc) for provision of safe, high-quality, patient-centered care focused on SDH | | |
| **Outcome/ EPA 1: Work Effectively with Physicians and Other Colleagues in the Health-care Professions to Improve SDH of Patients Presenting to the ED** | | |
| Identify personal strengths and weaknesses in maintaining professional relationships with others. | Negotiate overlapping and shared responsibilities with all colleagues in ongoing patient care. | Maintain positive relationships with all colleagues to support collaborative care in ED. |
| **Outcome/ EPA 2: Establish Liaison with Community Champions and Law Enforcement Agencies for Addressing SDH in ED** | | |
| Understand what lawyers & social workers can do for patients to address the social determinants of health. | Become aware of the available local resources/referrals for patients with legal needs. | Be able to connect patients to legal resources when appropriate. |
| **Health Advocate:**  The residents should efficiently use their expertise and influence to advance the health and well-being of individual patients, communities and populations by improving their SDH. | | |
| **Outcome/ EPA 1: To Consider the Ways in Which the Built Environment and Urban Design Affect Health Outcomes and Healthcare Utilization** | | |
| Understand built environment as man-made, physical attributes of our surroundings, including health-promoting resources and undesirable amenities that influence individual and community health behaviors. | Participate in advocacy activities needed for patients and families to directly impact health outcomes and health care utilization. | Develop creative solutions to mitigate the effects of the built environment and advocate for policies that support healthy built environments and environmental justice. |
| **Outcome/ EPA 2: Use Advocacy to Advance the Health of Individual Patients and Their Families** | | |
| Regularly practice health advocacy in ED by emphasizing behaviors that promote health and prevent disease. | Assist in planning health related activities in community in collaboration with social services. | Teach advocacy to junior residents, students and other healthcare providers. |
| The T **Professional:**  Residents should demonstrate habitual and judicious use of communication, knowledge, technical  skills, clinical reasoning, emotional and social values and reflection in daily practice of SDH for the  benefit of the individual and community being served.  The | | |
| **Outcome/ EPA1: Demonstrate Appropriate Professional Behavior in all Aspects of Practice** | | |
| Maintain good  interpersonal  relations with coworkers. | Show appropriate  manners in patient  interactions. | Demonstrate awareness of  limitation and seek  help. |
| **Outcome/EPA 2. Adhere to High Ethical Standards in Practice** | | |
| Acknowledge rights of  patients in clinical care. | Show commitment to  patient care. | Recognize, respond and manage  ethical issues in clinical  work. |
| **Leader:**  Residents must demonstrate an awareness of and responsiveness to the larger context and system of health care. Residents should acquire the ability to effectively lead within ED to provide optimal quality of care to their patients, and play their role in development of healthcare within the hospital, city and country to improve SDH and promote SEM. | | |
| **Outcome/ EPA 1: Engage with Other Healthcare Providers to Contribute to a High-Quality Healthcare System** | | |
| Analyze patient safety incidents to enhance system of care while addressing SDH. | Use clinical audit to improve the quality of patient care and optimize patient safety. | Contribute to a culture that promotes patient safety by participating in quality improvement activities & self-reflection. |
| **Outcome 2: Take Responsibility for the Delivery of Excellent Patient Care along with Health-care members** | | |
| Seek guidance and feedback from health care team members following interventions to improve patient SDH. | Communicate clearly with other team members regarding management and co-ordinate efforts of team members. | Take responsibility for following up on patient outcomes and ensure that the outcomes are checked in a timely manner. |

**Annexure 2: Thematic Breakdown of SEM Curriculum (Only one theme discussed)**

| **Theme: Health Literacy**  **EPA: Demonstrate the Importance of Healthcare Provider Communication in Improving Health Literacy for Patient** | | | |
| --- | --- | --- | --- |
| **Year wise Learning Objectives** | **Outcome** | **Teaching/ learning strategy** | **Assessment** |
| **Year 1-5**  Ensure that patients and their care givers understand medical instructions | -Medical expert  -Communicator  -HC advocate | -Tutorials  -Case based discussion  -Bedside teaching  -Simulation | -Mini CEX  -OSCE  -Chart stimulated recall (CSR) |
| **Year 1-5**  Identify the need of trained interpreter and mobilize resources | -Medical expert  -Collaborator  -Communicator | -Case based discussion  -Bedside teaching  -Role modelling  -Journal club | -Portfolio  -Mini-CEX  -OSCE  -CSR  -Multi-source feedback |
| **Year 1-5**  Ensure continuity of care by developing materials like pre-printed discharge instructions | -Medical expert  -Leader  -Scholar | -Case based discussion  -Bedside teaching  -Role modelling | -Portfolio  -Mini-CEX  -OSCE  -CSR  -Multi-source feedback |
